# Dynamic Heterogeneity in the Optical Signals from Single Nano-Objects

**DOI:** 10.1021/acs.jpcb.2c09055

**Published:** 2023-04-28

**Authors:** Michel Orrit

**Affiliations:** Huygens-Kamerlingh Onnes Laboratory, Leiden University, 2300 RA Leiden, Netherlands

## Abstract

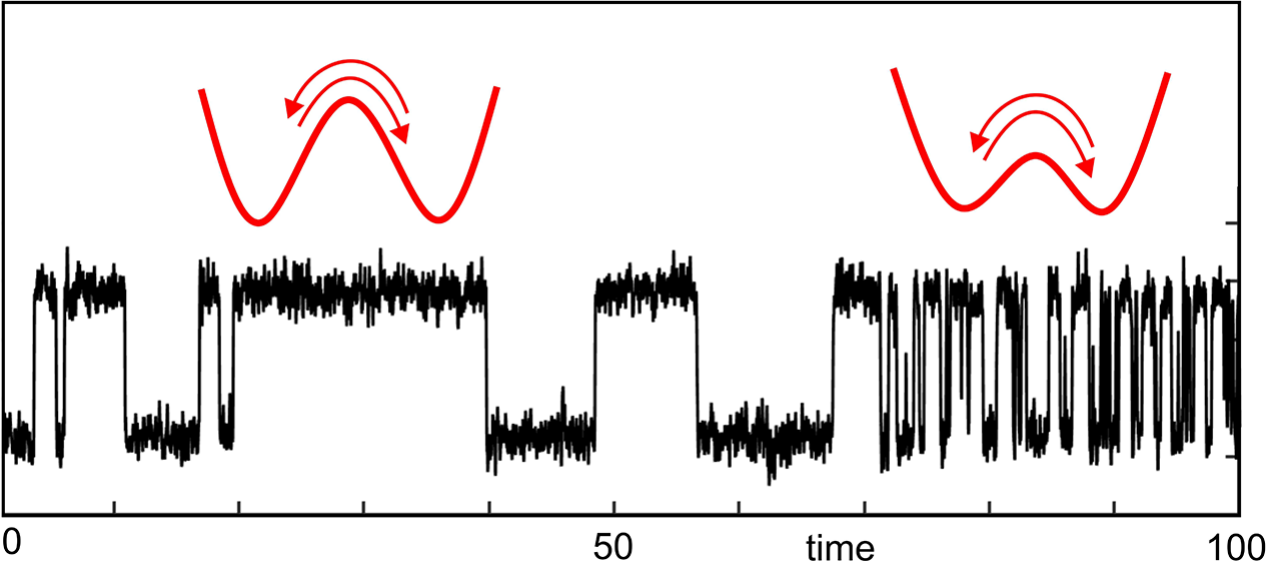

In contrast to ensemble-averaged measurements, single-molecule experiments directly display the heterogeneity of molecular properties in space and time. In many complex systems, spatial heterogeneity is regularly accompanied by temporal or dynamic heterogeneity; if a property differs from molecule to molecule, it will often vary in time for one and the same molecule. In this short paper, we discuss a few examples of complex systems where dynamical heterogeneity was observed in single-molecule or single-particle optical signals. For single biomolecules, the first demonstration of dynamic heterogeneity in a single enzyme was provided by Xie and colleagues. Other examples are found in glassy systems, and very recently in the magnetic relaxation of single superparamagnetic nanoparticles. The ubiquity of this phenomenon suggests that, rather than an exception, dynamic heterogeneity is the rule in complex systems with multiple degrees of freedom.

## 1 Introduction

The heterogeneity of complex systems such as glasses or proteins^1^ has been known for a long time and manifests itself in ensemble experiments through nonexponential relaxation rates and dispersive kinetics. But is nonexponentiality a mere consequence of differences from one local environment to another (say, differences from one molecule to the next), or would a measurement on a single molecule also show nonexponential kinetics? Would nonexponentiality at the single-molecule level reach the same extent as that of the ensemble? These questions usually cannot be answered by means of ensemble experiments, because averaging over all molecules erases any information about which molecules contribute to the averaged signal, and how they do it. Selective methods, such as persistent spectral hole-burning,^2^ provide some insight into static and dynamic heterogeneity, because they select a subpopulation of the ensemble, which is “tagged” and monitored as a function of time. Yet, selection by hole-burning still addresses a subensemble; i.e., it still includes a large number of molecules potentially differing through parameters other than their excitation energy. Single-molecule measurements, through which one and the same molecule can be followed over extended durations, provides the ultimate resolution and can answer the question of dynamical versus static heterogeneity. It provides the ultimate resolution in tagging and monitoring time-dependent properties, completely free from ensemble averaging. A watershed in studies of heterogeneity was the first investigation of single enzymes by Xie and colleagues in 1998,^1^ which forcefully demonstrated that both static and dynamic heterogeneity coexist in biomolecules.

Dynamical heterogeneity, or dynamical disorder as it was called in the early literature,^3^ is related to the property of ergodicity and the loss thereof, ergodicity breaking.^4^ Strong ergodicity breaking refers to a case of frozen or static heterogeneity, where subsystems never relax toward the average. Weak ergodicity breaking, in contrast, refers to subsystems that can fluctuate and relax toward the average, albeit on time scales which are longer—often by several orders of magnitude—than experimental observation times. Weak ergodicity breaking gives rise to what we understand by dynamical heterogeneity in this Perspective: a system’s dynamics which appear steady on a given time scale but may change rate or nature when the observation time is extended. To simplify the discussion of this intricate subject, let us consider a single molecular process characterized by a rate, for example, a reaction rate or a diffusion rate. For each molecule in its local environment, this rate can fluctuate in time in a dynamic manner. If the fluctuation rate is much larger than the reaction rate, any dynamical fluctuations will average out, so that the system will appear to be dynamically homogeneous.^5^ A good example is a simple liquid, where fluctuations are extremely fast, and where the reaction rates of all solute molecules are identical. If, on the other hand, the rate fluctuates extremely slowly compared to the process of interest and to measurement times, the heterogeneity may be considered as frozen or static. Think of a frozen glassy solution as an example, where each solute molecule has an individual but constant optical resonance frequency. In that case, the disorder is purely static, with no dynamic component. When the rate fluctuations of each molecule arise from variations of a single control parameter, either through jumps of this parameter between fixed values, or through its wandering under a generalized Langevin equation, analytical solutions can often be proposed, as discussed by Zwanzig.^3^ In general, however, fluctuations occur not only through one but also through many processes which span a very broad range of relaxation times, often covering many orders of magnitude. Such broad relaxation time scales span many orders of magnitude and commonly arise from the exponential dependency of reaction rates upon the height and width of the reaction barrier(s). Moreover, these control variables, far from being independent from each other, often are intricately coupled. As a consequence, the system is better described as wandering over a multidimensional potential (free-)energy landscape (PEL). Even a single molecule—be it a small molecule coupled to such a complex environment as a glass, or a biomolecule such as a protein, with its own complex PEL—will thus present characters of both static and dynamic heterogeneity.

As long as only ensemble-averaged measurements were available, the time- and space-averaged kinetics of complex systems could be described by effective laws, such as continuous-time random walks^6^ or time-dependent reaction rates.^7^ However, these effective descriptions are too coarse-grained to describe single-molecule experiments, which directly and for the first time have exhibited the general coexistence of static and dynamic heterogeneity in many very different systems. The aim of this short paper is to focus on four examples of systems with dynamic heterogeneity and to reflect about their underlying properties. This choice leaves aside many other systems, such as intermittent photoluminescence time traces of single quantum dots,^8^ which can also display dynamical heterogeneity.

(i) Proteins at physiological or ambient conditions notoriously display nonexponential reaction rates. The most classical example is the CO-rebinding reaction in myoglobin, analyzed in ensemble experiments by Frauenfelder and colleagues.^9^ At about 100–200 K, the rate of ligand rebinding after flash photolysis is distributed over many decades, from microseconds to minutes and beyond. The distribution of rates was assigned to static disorder, but no single-molecule experiment has yet clarified the extent of dynamic disorder in this reaction. The first direct detection of both static and dynamic heterogeneity of a single protein was reported in 1998 by Xie’s group, in autofluorescent cholesterol oxidase.^1^ Similar observations have been reported many times since then.^10−13^ The presence of static and dynamic heterogeneity, even in comparatively small proteins is nowadays well documented.
(ii) The physical properties of ill-condensed solids, notably glasses and polymers, at very low temperatures are often described phenomenologically by two-level systems (TLSs). Single-molecule experiments not only confirmed the reality of those two-level systems^14^ but also, in many cases, revealed their mutual couplings and interactions.^15^
(iii) At significantly higher temperatures, glassy and polymeric systems undergo a glass transition, a kinetic transition below which extremely slow relaxation processes appear. The relaxation times of these degrees of freedom are so long that, for all practical purposes, they appear frozen, giving rise to static heterogeneity. Relaxation of these systems is much too complex to be described by TLS models. However, small local probes such as single molecules can experience very heterogeneous local environments and reveal dynamic as well as static heterogeneity.^16^
(iv) Yet another example of dynamic heterogeneity has recently been found in the magnetic relaxation of single magnetite nanoparticles,^17^ to which complex magnetic energy landscapes can be associated.^18^

All these examples suggest that dynamical heterogeneity, far from being a rarity, is rather the rule than the exception in complex many-body systems and is usually associated with static heterogeneity.

## 2 Results

### 2.1 Proteins

As mentioned earlier, one of the most convincing evidences for heterogeneity in protein dynamics came from the CO-rebinding experiments in myoglobin by Frauenfelder and colleagues.^9^ However, as those measurements were ensemble-averaged, it was impossible to assign the observed stretched kinetics to static or dynamic heterogeneity. The optical isolation of single-molecule fluorescence signals made it possible to follow a single protein molecules over extended periods of time, thereby establishing robust statistics of reaction rates whenever the reaction events could be identified through fluorescence. Using the autofluorescence of the flavin cofactor of cholesterol oxidase, Lu et al.^1^ published the first report of *single-molecule* static and dynamic heterogeneity. Not only did the turnover rate of the enzyme vary from molecule to molecule, revealing static heterogeneities, the turnover rate of a single enzyme fluctuated over accumulation times limited by photobleaching of the flavin, typically seconds. These times corresponded to several turnovers of the enzymatic reaction and indicated that the protein was undergoing conformational changes over the same time scales. Such observations are not compatible with the standard Michaelis–Menten model, with its constant rates, although the same kinetics law can be recovered with effective rate parameters.^20^ In the same group, Yang et al.^21^ investigated the lifetime of the FAD cofactor of another enzyme, flavin reductase. Electron transfer from the optically excited FAD toward an adjacent tyrosine residue reduced the fluorescence lifetime of flavin. Considerable lifetime fluctuations were therefore assigned to fluctuations of the electron transfer rate, themselves ascribed to temporal variations in the distance between flavin and tyrosine. The time traces of fluorescence lifetimes present very direct and convincing evidence of dynamical heterogeneity of single flavin reductase enzymes, due to subtle conformational rearrangements of the molecules.

Later work by other authors confirmed the generality of these initial observations. Hofkens and colleagues reported direct observations of lipase turnovers on a lipid monolayer over extended times of the order of hours, with a fluorogenic reaction generating a fluorescent product.^22^ Dividing the fluorescence trace into on- and off-levels, the authors monitored the enzyme’s trajectory and plotted waiting time distributions following stretched exponentials. The results supported the model of a fluctuating enzyme, with conformational changes occurring over a broad range of times much exceeding those of the catalytic reaction, and generating “lazy” and “busy” times for a single molecule. It should be noted that the diffusion of the fluorescent products around the enzyme complicates the analysis of the data. Moreover, as has been later realized,^23^ the statistical tools used to analyze the data, particularly when thresholds are used, can easily introduce artifacts. Experiments by Rigler and colleagues^10^ on horseradish peroxidase, in accordance with previously reported results, supported the hypothesis that slow conformational fluctuations of the enzymes influence their catalytic activity. Another electron-transfer protein, quiescin sulfhydryl oxidase (QSOX), exhibits stretched power-law kinetics of its open-closed dynamics. Single-pair FRET experiments from Hofmann’s group^24^ spanning times from nanoseconds to milliseconds, suggested that this kinetics arises from exploration of an ensemble of disordered domain orientations, corresponding to the rugged PEL postulated in ensemble experiments, here for a multidomain enzyme.

**Figure 1:**
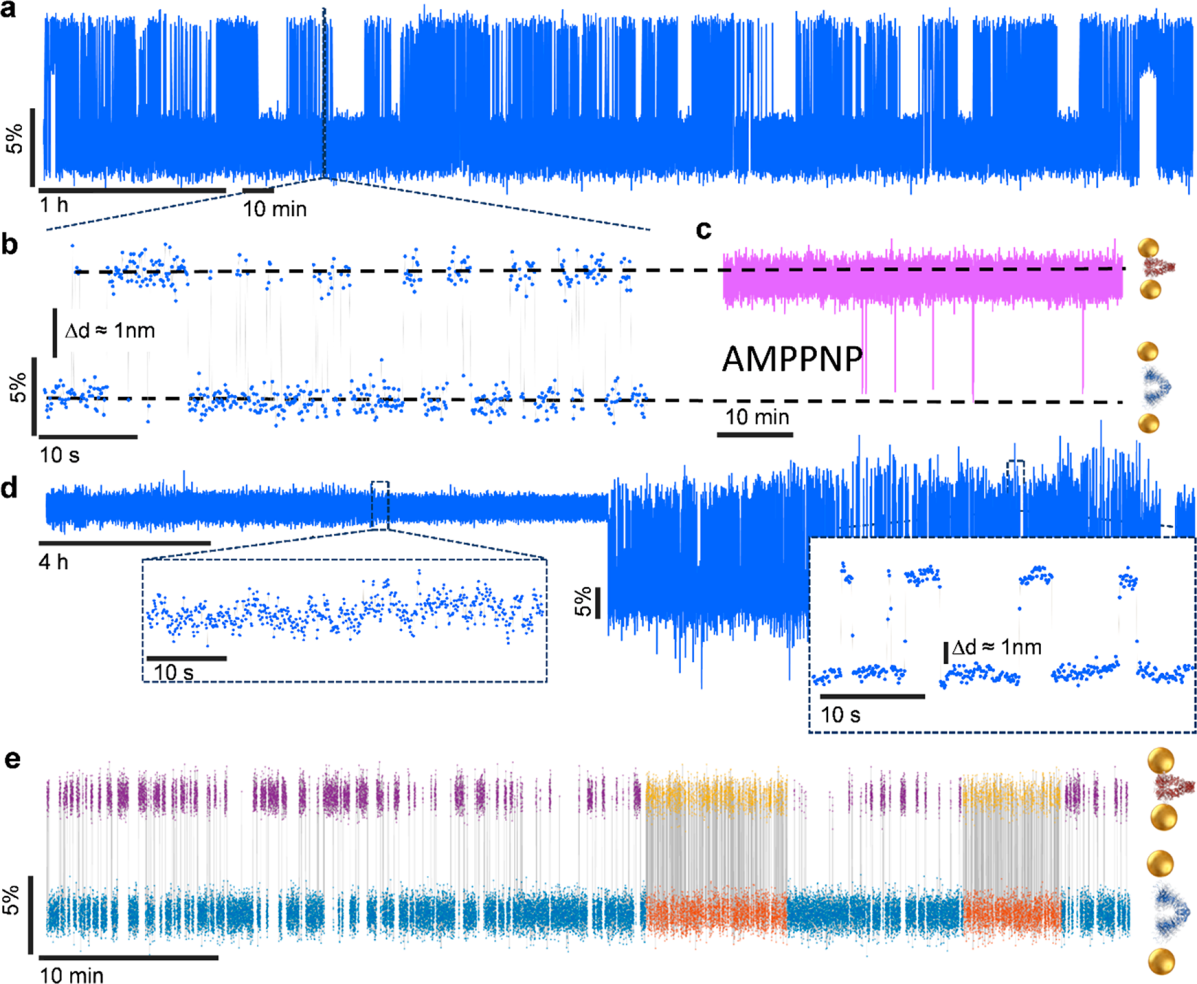
(a) Time trace of the light scattering signal of a single Hsp90 protein molecule carrying two gold nanoparticles acting as a plasmon ruler. The scattering spectrum shifts according to the distance between the particles and thereby reports on the protein’s jumps between two conformations, open and closed, characterized by different scattering signals. (a) The photostability of gold particles enables recording of very long time traces (here 6 h, with a time resolution of 100 ms), which display striking examples of dynamical heterogeneity. Note the alternation between frozen and active periods. The frozen periods occur mostly in the open conformation but also in the closed one toward the end of the trace. (b) Zoom-in of one of the active periods. (c) Addition of a nonhydrolyzable ATP-analogue (AMPPNP) blocks the protein in the open conformation. (d) Another trace, recorded for 24 h at 50 ms resolution, showing the transition from blocked to active state. (e) Third time trace showing fluctuations of the transition rate itself. Reproduced with permission from ref (19). Copyright 2018 American Chemical Society.

**Figure 2:**
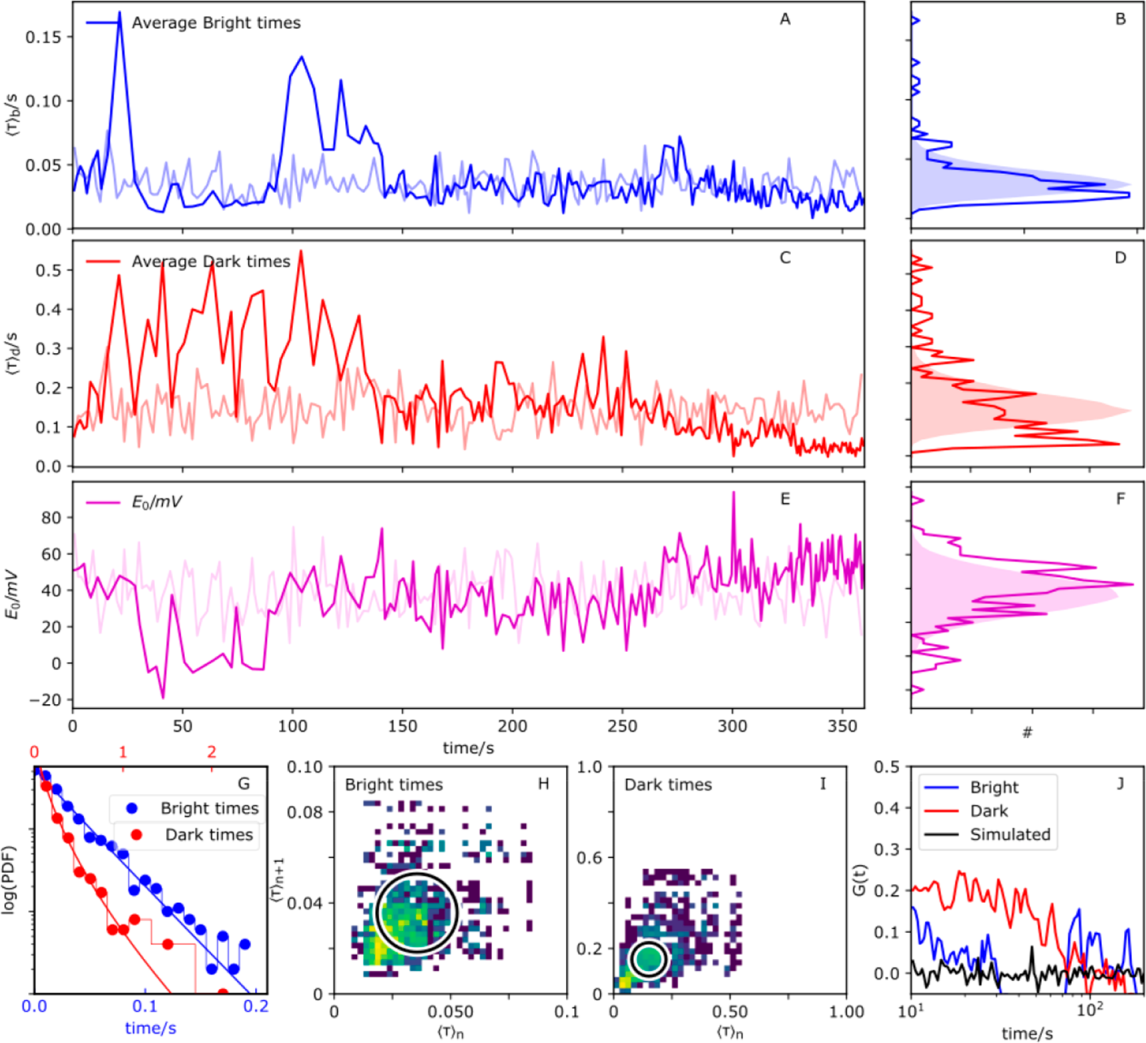
Data extracted from a time trace of a single azurin molecule undergoing redox turnover cycles at a controlled redox potential. Panels A, C: Averages of ten consecutive bright (A: reduced state) and dark (C: oxidized state) times are plotted as a function of the measurement time. Histograms of these averages are plotted in panels B and D and compared to histograms from simulated traces (shaded profiles) where the switching rates are kept constant. Deviations of the histograms from the shaded profiles show excesses of consecutive shorter or longer average times. Alternatively, consecutive average times are correlated against each other in panels H and I. The circled areas indicate where uncorrelated data should be found with 95% probability in the absence of heterogeneity. The large fraction of points outside these circles is a direct visualization of dynamic disorder. Panel G shows the distribution of times, and panel J, their autocorrelation function. Reproduced from ref (13) under CC-BY license terms. Copyright 2020 Royal Society of Chemistry.

Long time traces of single proteins (heat shock protein Hsp90) were recorded in Soennichsen’s group^19^ thanks to a plasmonic ruler. The plasmon resonance of a set of two gold nanoparticles attached to the same protein was recorded continuously over extremely long times. The optical scattering signal is free from bleaching and fluctuates according to the distance between the particles, itself a function of protein conformation. As displayed in the example of Figure 1, these time traces show spectacular slow-downs of protein activity indicative of extreme dynamic heterogeneity. Surprisingly, dynamic heterogeneity is also found in small proteins with a robust well-defined structure, as the example of azurin shows. Pradhan et al.^13^ studied long time traces of single fluorescently labeled azurin molecules, where the fluorescence is quenched in the oxidized state of the copper center, whereas it is emitted unimpeded in the reduced state. The single electron transfer turnovers can then be identified in fluorescence time traces and again show fluctuations of the electron transfer rate attributed to conformational changes (see Figure 2). Those multiple observations in very different molecules, from simple to complex, support the general argument that the high complexity and multidimensionality of a protein’s free energy landscape is the underlying cause of dynamical heterogeneity, which should be common, if not universal, for many proteins, including those with a well-defined fold and structure.

The example of fluorogenic reactions shows the importance of robust statistics and user-independent evaluation algorithms in the search for heterogeneity. In ref (1), turnover times of successive events were correlated with one another and showed deviations from the expected scatter plots from uncorrelated events.^25^ However, the distribution of turnover times is subject to Poisson fluctuations. To reduce these fluctuations, Pradhan et al.^13^ have averaged a number of consecutive turnover times (ten of them) and correlated these averaged times in the way used earlier for single times (see examples in Figure 2H,I). The reduced spread of these averages highlights changes of the average with time, at the expense of the time resolution with which such changes can be detected with high statistical confidence.

### 2.2 Disordered Solids at Cryogenic Temperature

At cryogenic temperatures, disordered solids such as glasses and polymers present excess heat capacity and ultrasound absorption arising from the activation of low-energy degrees of freedom. These excitations are attributed to local rearrangements of atoms or groups of atoms in small regions and are often modeled as nearly independent two-level systems (TLSs), as long as the temperature is low enough, typically below a few degrees Kelvin. The rate of flipping of TLSs depends exponentially on barrier parameters, whether the process is classical activation or quantum-mechanical tunnelling between the two wells. Therefore, some TLSs are exponentially slow, and their jump rates will always exceed experimental measurement scales. Such disordered systems will thus always present some degree of static heterogeneity.

When single fluorescent molecules are dispersed in such polymers and glasses at cryogenic conditions, they often present very sharp optical lines, which become ultrasensitive reporters of the local conditions. These lines couple to TLSs through electrostatic or elastic interactions and are often observed to give rise to spectral jumps of single-molecule lines between two positions,^14^ in good agreement with the TLS model. In some cases, the lines are found to jump between 4 or 8 positions, and such jumps are assigned to independent flips of respectively 2 or 3 TLSs in the molecule’s environment. Indeed, these spectral shifts are often found to be additive, in agreement with the simplest hypothesis of independent TLSs with additive interactions with the single molecule under study. In a significant fraction of the molecules studied, however, the jumps were not additive,^15^ which means that a jump of a slow TLS (TLS1) may modify the jump rate or the jump amplitude of a faster TLS (TLS2). In other words, the PEL of the glass for TLS2 may change shape according to the state in which TLS1 is currently residing. Therefore, these TLSs are not independent, they are coupled. This coupling leads to dynamical heterogeneity, as the slow degree of freedom of TLS1 enslaves the faster one of TLS2.^26^ When temperature is increased and the number of activated TLSs thereby effectively increases, the probability of finding nearby, thus interacting, TLSs will increase. This example of a relatively simple multidimensional disordered system shows how dynamics can continuously change. The glass heterogeneity is purely static at low temperature, when each molecule either does not jump or jumps at a constant rate or at a small number of constant rates. As the temperature is raised, the glass acquires dynamic in addition to static heterogeneity, and the environment of each molecule starts to explore the multidimensional PEL with more and more complex patterns of jumps. In the next section, also devoted to the glassy phase, we approach it from the other end, cooling a liquid from temperatures higher than its glass transition.

### 2.3 Glasses and Supercooled Liquids

When a liquid is cooled below its glass transition temperature, while avoiding crystallization, some of its relaxation times become extremely long, longer than experimental time scales. This observation is captured by the mode-coupling theory of the glass transition. The appearance of very long relaxation times means that density fluctuations in the supercooled liquid may appear frozen on measurement time scales. Therefore, static heterogeneity will be observed in measurements of the rotational diffusion of single probe molecules dispersed in the system.^16,27−30^ Different molecules at different locations in the fluid are found to tumble at different rates, indicating variations of the local viscosity of the supercooled liquid. This heterogeneity can be interpreted as variations in the local concentration of voids in the glass-forming material and may be thought of schematically as different liquid-like ponds separated by solid-like walls.^28,31^ The walls themselves may evolve or relax over time, so that the local viscosity may change, thereby modifying the rate of rotational diffusion of probe molecules. Single molecules will thus exhibit dynamic heterogeneity. Moreover, as the probe molecule itself may translationally diffuse from one to another pond, it may experience a change in local mobility, again appearing as dynamic heterogeneity. These events of changes in viscosity are called “exchanges” in the glass dynamics literature.^16^ They may be nearly as short as the rotational diffusion times themselves (shorter exchanges would not be observable) but can also become extremely long when the system is allowed to age over extended periods of time.^28,31^ A more extensive review of heterogeneity in supercooled liquids can be found in ref (32).

### 2.4 Magnetic Relaxation of Single Nanoparticles

A very different kind of complex systems is magnetic nanoparticles and nanostructures, which are often modeled as presenting a single macro-spin, where all the particle’s spins remain parallel to one another due to strong exchange interactions but collectively switch “en bloc” from one orientation to another one. The Néel–Brown model of superparamagnetism^33^ considers only two potential wells for the PEL of the macro-spin, both aligned with the long axis of the particle^34^ but with opposite directions. In this model, the particle is considered as ferromagnetic on the one hand if the magnetization is frozen in one orientation and does not change over the experimental measurement time. This will be the case of large particles at low temperatures. If, on the other hand, the particle is small enough or the temperature high enough, the magnetization will be flipping back and forth under the influence of thermal fluctuations, with flipping times shorter than the observation time.^35,36^ Such a particle is said to be superparamagnetic. In fact, the Néel–Brown PEL is a dramatic simplification. When the many degrees of freedom of the particle’s individual constituting spins are considered to vary independently from one another, the associated multidimensional PEL can present a much more complex shape with local minima and saddle points,^18,100^ which may give rise to static and dynamic heterogeneity.

**Figure 3:**
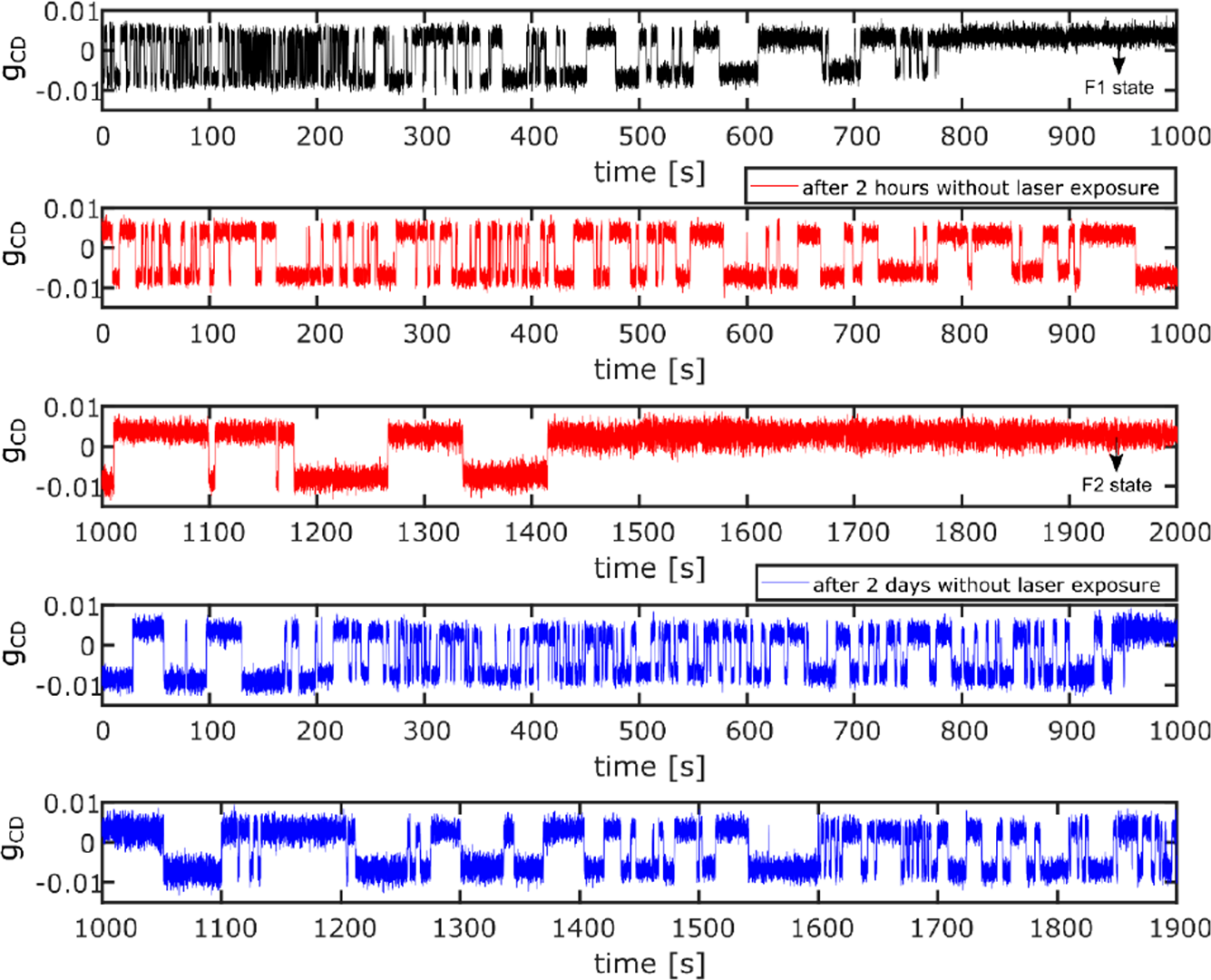
Time traces of the optical photothermal signal of the same single magnetite nanoparticle, 20 nm in diameter. The polarization of the excitation light is modulated at high frequency, and the ensuing small temperature change reveals a small change of absorption (g_CD_), which itself reports on the orientation of the particle’s magnetization. The magnetization is seen to flip randomly on time scales of seconds. The time traces have been recorded at different times, spaced by up to 2 days. Obvious changes in the statistics of these flips display evidence for dynamic heterogeneity; reproduced from ref (17) under CC-BY license terms. Copyright 2022 The Authors.

Adhikari et al.^17^ have recently recorded long time traces of the magnetization of individual magnetite nanoparticles 20 nm in diameter by a photothermal method involving the polar magneto-optical Kerr effect. Indeed, some particles were found to switch magnetization between two opposite values during the measurement of the time trace. Figure 3 shows examples of magnetization time traces of the *same* nanoparticle at different times. Clearly, the switching rate changes during each time trace (see in particular the upper trace), indicating a clear case of dynamic heterogeneity. Such rate variations would obviously not occur in the Néel–Brown model, as the switching barrier would remain constant in time. Changes of the switching rate indicate some influence of slow fluctuations of unknown control parameter(s), which cause fluctuations of the barrier height and thus typical displays of dynamic heterogeneity. The nature of these exchanges is still open to speculation. They might be due to surface reactions with ligands, or to local fluctuations of the particle’s stoichiometry in iron-II and iron-III, perhaps accompanied by structural reorganization.

## 3 Discussion and Conclusion

We have encountered heterogeneity in systems with increasing complexity. Starting from a glass at very low temperature, with a low concentration of flipping two-level systems, we found static heterogeneity. As the two-level systems are few and far between, they are not very likely to be coupled yet, and the dynamics we find are homogeneous in time, apart from a few occasional spectral jumps when a TLS flips. When the temperature is raised, however, more TLSs get activated, and coupling between TLSs becomes more and more common. The spectral dynamics due to fast TLSs changes according to flips of the slower TLSs.

Approaching the glassy phase from the other end, we start from a simple molecular liquid, which is dynamically homogeneous, at least on the experimental time scale of fluorescence measurements, microseconds and longer. In such a liquid, all solvent molecules behave in the same way on average, just as solute molecules do. However, as soon as temperature is lowered and very slow fluctuation modes appear, in the vicinity of the glass transition temperature, the range of relaxation times broadens and extends to infinity. Because of coupling between local rearranging regions, mediated by the embedding material, we expect dynamical heterogeneity to become prominent. If we monitor diffusing molecular probes in a glass-forming material, the heterogeneity of local environments is compounded by the possibility for the probes to explore different local environments in turn.

It may not be too surprising to find heterogeneity in an essentially infinite system such as a polymer or a supercooled glassy liquid, but what about smaller systems such as single, isolated nanoparticles and biomolecules? How small must a particle be for all its degrees of freedom to be faster than experimental measurement times? Would its dynamics then appear homogeneous from a dynamical but also from a static point of view? We have seen that magnetite particles of around a million atoms present very clear dynamical heterogeneity of their magnetic relaxation. Azurin, a small protein with a mass of only 14 kDa (128 residues) also presents static and dynamic heterogeneity. We may therefore speculate that any protein, even one with a well-defined fold, may present static and dynamic heterogeneity. On the one hand, long-range interactions responsible for allostery^11^ may favor long-range coupling and thus favor the occurrence of heterogeneity in proteins compared to other noncrystalline nanoparticles. On the other hand, intrinsically disordered proteins (IDPs) might explore their whole configuration space during experimental measurement times, averaging out static and dynamic heterogeneity.

Our short overview indicates that dynamical heterogeneity is common in a broad variety of complex systems. The ingredients required for its presence appear to be

(i) multiple degrees of freedom and a multidimensional PEL;
(ii) a broad distribution of relaxation times, particularly extending to durations much longer than the measurement, so that even though part of the degrees of freedom are frozen, many of them are still active on a broad range of time scales;
(iii) a significant degree of coupling between these degrees of freedom, so that slow ones can enslave faster ones and local dynamics change character in time. This coupling requires nonadditive contributions to energy and is expected to be particularly important in proteins, which have been selected for the interdependence of their degrees of freedom making allostery possible.

Based on this tentative characterization, in which systems could we expect to find dynamical heterogeneity? A few examples come to mind:

(i) Catalysts beyond enzymes present active sites which are very sensitive to structure.^37^ Subtle rearrangements around catalytic sites would lead to heterogeneity in time as well as in space, in full analogy to biological enzymes.
(ii) Disordered conductors, such as organic solids^38^ or conjugated polymers^39^ have complex mechanisms and pathways for the transport of charge carriers or excitons. The distribution of trapped charges in semiconductor quantum dots or in perovskite nanocrystals^8,40^ is believed to be responsible for their complex optical properties, including luminescence blinking.
(iii) For the translational or rotational diffusion of molecules in composite and porous materials, the network of accessible pathways introduces long-range couplings that may be expected to cause dynamic heterogeneity.

In conclusion, therefore, dynamical heterogeneity appears to be the rule for complex systems with long relaxation times, rather than an exceptional property confined to a few examples. Just pointing out dynamical heterogeneity in a system is thus not particularly informative. The really interesting but much more difficult question will be to understand which potential energy landscapes give rise to dynamical heterogeneity and which parameters control the landscape’s shape and topography. Solving these new questions will require full-fledged investigations of the dynamics of these systems. Single-molecule microscopy appears the method of choice to provide those answers in the coming years.
